# Transcription start sites and epigenetic analysis of the *HSD17B10* proximal promoter

**DOI:** 10.1186/1471-2091-14-17

**Published:** 2013-07-08

**Authors:** Song-Yu Yang, Carl Dobkin, Xue-Ying He, W Ted Brown

**Affiliations:** 1 Department of Developmental Biochemistry, NYS Institute for Basic Research in Developmental Disabilities, 1050 Forest Hill Road, Staten Island, NY 10314, USA; 2 Department of Human Genetics, NYS Institute for Basic Research in Developmental Disabilities, 1050 Forest Hill Road, Staten Island, NY 10314, USA

**Keywords:** CpG island, DNA methylation, TATA-less promoter, X-chromosome inactivation, HSD10 deficiency

## Abstract

**Background:**

Hydroxysteroid (17beta) dehydrogenase X (HSD10) is a multifunctional protein encoded by the *HSD17B10* gene at Xp11.2. In response to stress or hypoxia-ischemia its levels increase rapidly. Expression of this gene is also elevated significantly in colonic mucosa of the inactive ulcerative colitis patients. However, accurate information about its several transcripts is still lacking, and additional evidence for its escape from X-chromosome inactivation remains to be obtained in order to help settle a debate (He XY, Dobkin C, Yang SY: Does the *HSD17B10* gene escape from X-inactivation? *Eur J Hum Genet* 2011, **19**: 123-124).

**Results:**

Two major *HSD17B10* transcription start sites were identified by primer extension at -37 and -6 as well as a minor start site at -12 nucleotides from the initiation codon ATG. Epigenetic analysis of the 5’-flanking region of the *HSD17B10* gene showed that there was little 5-methylcytosine (<3%) in a normal male, and that none of CpG dinucleotides in the CpG island approached 50% methylation in females.

**Conclusion:**

The actual length of first exon of the *HSD17B10* gene was found to be about a quarter larger than that originally reported. Its transcripts result from a slippery transcription complex. The hypomethylation of the CpG island provides additional evidence for the variable escape of the *HSD17B10* gene from X-chromosome inactivation which could influence the range of severity of HSD10 deficiency, an inherited error in isoleucine metabolism, in heterozygous females.

## Background

Hydroxysteroid (17beta) dehydrogenase X (HSD10) is encoded by the *HSD17B10* gene at Xp11.2 [1]. HSD10 is a multifunctional enzyme involved in the degradation of isoleucine and branched-chain fatty acids, the metabolism of steroid hormones and neuroactive steroids as well as aldehyde detoxification [2,3]. Furthermore, it complexes with other proteins to generate RNase P activity [4]. Various HSD10 functions are inhibited when it is bound to the amyloid-β peptide [5] or estrogen receptor alpha [6].

Mutations in the *HSD17B10* gene and its aberrant expression result in HSD10 deficiency (OMIM#300438) [7,8], X-linked mental retardation, and abnormal behavior (MRXS10) (OMIM#300220) [9]. Accumulation of HSD10 in neurons is also involved in the pathogenesis of Alzheimer disease (AD) [10]. Elevated levels of HSD10 are found in the cerebrospinal fluid (CSF) of patients with AD and multiple sclerosis [11]. HSD10 levels vary significantly among different brain regions and in response to stress and hypo-ischemia [12,13]. The expression of the *HSD17B10* gene was also significantly elevated in colonic mucosa of the inactive ulcerative colitis patients [14,15]. In order to lay the foundation for designing effective treatment of these disease conditions, it is essential to elucidate the molecular mechanism of the *HSD17B10* gene’s expression. For this reason we sought accurate information about its transcripts and DNA methylation of its CpG island.

Here we report that there are several transcription start sites. The predominant transcript, HSD10 variant 1, starts at -37 or -6 nucleotides from the ATG initiation codon. In addition we found that none of CpG dinucleotides in the in the *HSD17B10* proximal promoter CpG island region, approached 50% methylation in female genomic DNA. Since X inactivation of *HSD17B10* would be expected to result in nearly complete methylation of this region in one of the two copies of this gene, this hypomethylation of the CpG island, together with previously reported data of *HSD17B10* expression in somatic cell hybrids [16], suggests that this gene may have a variable escape from X-chromosome inactivation.

## Methods

### Ethics statement

This study was approved by the Institutional Review Board of NYS Institute for Basic Research in Developmental Disabilities. The human DNA samples were obtained in conformance with the Internal Review Board’s guidelines and included the acquisition of written informed consent.

### Chromosomal DNA

Chromosomal DNA was isolated from blood samples of normal individuals (one male and two females) with the FlexiGene kit (http://www.qiagen.com) and used as the template for the *HSD17B10* gene-specific methylation analysis.

### Primer extension analysis

Total human brain RNA was purchased from Clontech. A primer HBHADPE3 (5’-CAGGTCCAGAAGCACAGCAGAGGCT-3’) specific to *HSD17B10*[17] was synthesized. This primer and phiX174 DNA/Hinf1 marker obtained from Promega were 5’-labeled, respectively, by using [γ-^32^P]ATP and T4 polynucleotide kinase as previously described [18]. 25μg human brain RNA mixed with 1 pmol of ^32^P-labeled primer in 17μl of buffer containing 75 mM KOAc, 50 mM Tris-HCL (pH 8.3), 8 mM Mg(OAc)_2,_ and 10 mM DTT was denatured in a heating block set at 100°C for 2 min, and then cooled quickly on ice. This sample was transferred to a 42°C water bath for hybridization for at least 30 min. During the annealing reaction, the sample was mixed with 2 μl dNTP (2mM each of dATP, dGTP, dTTP and 10 μCi [α-^32^P]dCTP) and 1 μl of AMV reverse transcriptase (20 units). Finally, 2 mM dCTP was added and the extension reaction proceeded at 42°C for another 30 min, as previously described in principle [19]. Three parallel experiments, either without RNA or without primer or without reverse transcriptase, were performed as controls. At the end of the primer extension, 4 μl of 0.5 M EDTA was added to stop the reaction. After the nucleic acid was recovered by ethanol precipitation, the pellet was resuspended in 6 μl of formamide gel loading buffer. Primer extension products were separated by electrophoresis on an 8% polyacrylamide gel in parallel with the [γ-^32^P]-labeled phiX174 DNA/Hinf1 marker and a dideoxy sequencing ladder from the dideoxy sequencing of a plasmid containing the *HSD17B10* 5’-flanking region ligated to the same gene's coding sequence.

### Bisulfite sequencing

Bisulfite modification and pyrosequencing analyses [20] of a 179 bp segment of the 5’-flanking region of the *HSD17B10* gene was done by EpigenDx, Inc. (http://www.epigendx.com) using the Zymo Research EZ methylation kit (http://www.zymoresearch.com), Hotstar Taq polymerase (http://www.qiagen.com), the PSQ™96HS system, and EpigenDx *HSD17B10* methylation assay kit (ADS2502FS). For experimental details the ASSAY DESIGN REPORT of the EpigenDx Inc. is included as the Additional file 1. The target sequence and pyrosequencing analysis are listed in Table 1.

**Table 1:** Methylation assay target sequences

**Assay ID**	**Genomic target sequence**	**Bisulfite converted target sequence**	**Pyrosequencing dispensation order**
ADS2502FS1	caggcggaatccgccctctggccaaaggactagcgtaccagg	taggyggaattygttttttggttaaaggattagygtattagg	ATCATGTCGTATCGTCTGCTAGACTATGTCGTA
ADS2502FS1	ccacgccccacgtctcatgcggcagcggcagacgccccggcccgtcgatccgccccttccgccgcttcgcctcggccaatcaacgagcgcccgcgcccccatcCCCATCCCGTGGAGTGGCCGGCGACAAGATGG (c>g, polymorphism rs1264014)	ttaygtttttaygttttatgyggtagyggtagaygtttyggttygtygtattygtttttttygtygt/gttygtttyggttaattaaygagygagygttygygttttattTTTATTTYGTGGAGTGGTYGGYGATAAGATGG	TCGATCGTCTGATCGTCTATAGTCGTCATGTCGTAGTATCAGTTCGTCAGTCGTCGATCGTTCAGTCGTTCTGTTCGTCATCGATCGATGTCAGTCGTCGTCTATTGATTCGTGAGTAGTCGTCGA

## Results

### Multiple transcription start sites of *HSD17B10* gene

**Figure 1:**
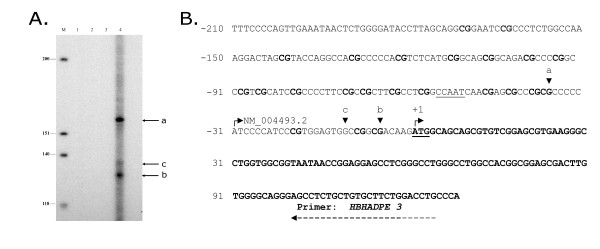
**Transcription start sites of human HSD17B10 gene were determined by primer extension.** Part **A**: (M), DNA molecular standards of which the sizes (bp) are indicated; (1), controls run without reverse transcriptase; (2), without RNA; (3), without primer; and (4), extension reaction mixture analyzed in parallel on an 8% polyacrylamide gel. The dideoxy sequencing ladder is included in the Additional file 2. Part **B**: Two major transcription start sites at -37 and -6, respectively, and a minor one at -12 are indicated by vertical arrowheads a, b, and c, respectively. CpG dinucleotides in the proximal promoter and the HSD17B10 cDNA nucleotide sequence are in bold. The oligonucleotide primer used in this study was complementary to a fragment of the HSD17B10 cDNA nucleotide sequence, and is shown by a long dash arrow. Both the initiation codon ATG, numbered as +1, and the CCAAT box are underlined.

The primer used for primer extension is complementary to the *HSD17B10* cDNA 102 to 126 nucleotides downstream from the ATG initiation codon. The results of a representative analysis are presented in Figure 1. The sizes of two prominent bands (a and b) show that two major transcription start sites are located in the 5’-flanking region of the *HSD17B10* gene at -37 and -6 base pairs upstream from the ATG initiation codon. A minor band (c) corresponds to an additional transcription start site at -12 upstream from the ATG, exactly matching the start position of a known HSD10 transcript HIT000033911. Also bands (b) and (c) correspond closely to the transcription start sites displayed in the ENCODE project [21] but both are one nucleotide further upstream.

### Hypomethylation of the CpG island in 5’-fflanking region of the *HSD17B10* gene

**Figure 2:**
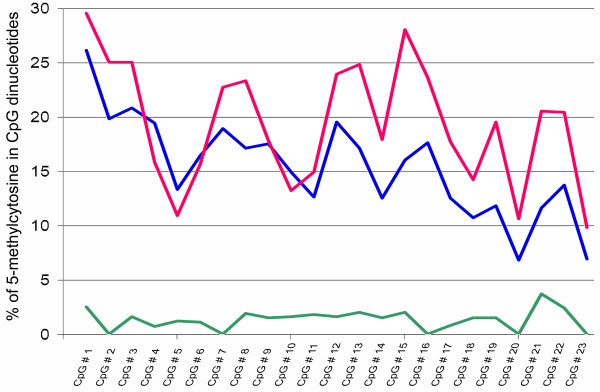
**Methylation analysis of the proximal promoter of HSD17B10 gene.** The percentage of 5-methylcytosines in different CpG dinucleotides is shown on the vertical axis. The nucleotide position for individual CpG dinucleotides can be found in Table 1 in the “Genomic Target Sequence” column. Red and blue lines represent the results obtained from two normal females while the green line shows the results from a normal male.

The 5’-flanking region of the *HSD17B10* gene contains a CpG island, consisting of 23 CpG dinucleotides in a region from -171 to -6 bp upstream from the ATG initiation condon (Table 1). An epigenetic analysis was performed for the *HSD17B10* proximal promoter. As expected few (<3%) of cytosines in CpGs of this promoter were found to be 5-methylated in male DNA (Figure 2). On the other hand, about 20% of cytosines in CpG dinucleotides were determined to be 5-methylated in females. In contrast with other genes subject to X-chromosome inactivation (XCI) [21], the *HSD17B10* proximal promoter was found not to be hypermethylated: none of CpG dinucleotides in this region approached 50% C5-methylation in the female DNAs analyzed.

## Discussion

HSD10 which is encoded by the *HSD17B10* gene [17], has both enzymatic and non-enzymatic functions that are central to a number of developmental disabilities and AD [22]. The *HSD17B10* proximal promoter has a CCAAT box (-56/-52 from ATG), but no TATA box (Figure 1B). So far fourteen different types of hydroxysteroid (17β) dehydrogenases have been identified in mammalian cells [23]. Genes encoding different types of hydroxysteroid (17β) except that for the type 1 hydroxysteroid (17β) dehydrogenase (HSD17B1), have a binding site for CCAAT enhancer binding factors (C/EBPs) in their promoters. A study focused on the impact of this family of transcription factors on gene expression in a hepatocarcinoma cell line HepG2 found that both *HSD17B8* and *HSD17B10* were regulated by C/EBPβ [24].

Three splice variants of HSD10 have been reported [1]. Since the ratio of HSD10 mRNA isoform 1 versus those of 2 and 3 is 3280:1:52 [1], the transcription start sites identified in this study are most likely related to the transcript of HSD10 variant 1 and probably to 2 and 3 as well, although this remains to be determined.

It was reported that there is approximately a 5-fold increase of HSD10 antigen in cortical neurons in the region surrounding cerebral ischemia consequent to stroke [13]. This observation suggest that the *HSD17B10* may be a hypoxia-responsive gene. Moreover, a significant change of *HSD17B10* expression was found in the colonic mucosa of patients with ulcerative colitis [14,15]. We examined alternative transcription start sites because alternative transcription start site usage has been linked to disease [25]. The first major transcription start site in human brain was identified 37 nucleotides upstream from the ATG initiation codon (see “a” in Figure 1B). This is 6 nucleotides upstream from the start of Ref. Seq NM_004493.2 (Figure 1B), which is itself 6 nucleotides upstream from the start site in the sequence we originally deposited to GenBank (AF035555.1) in 1997. The results indicate that the actual length of first exon of this gene is about one and a quarter of that reported previously [17]. The multiple transcription start sites identified here not only locate the RNA polymerase II binding sites but also suggest that the transcription complex may shift within this promoter.

The *HSD17B10* promoter qualifies as a high CpG density (HC) promoter (see Table 1), which is often associated with housekeeping genes [26]. The association between promoter methylation and transcriptional silencing is well established and promoter methylation is a reliable epigenetic mark of XCI [27]. The methylation analysis of a CpG island that overlaps a promoter, along with measurements of the relative expression of each allele identified by SNPs or expression analysis in somatic cell hybrids [16,28,29], is one of the most useful methods to predict the genes that escape XCI [30]. The epigenetic analysis of the *HSD17B10* proximal promoter showed that it is unmethylated in male DNA (<3% 5-methylcytosine) as expected, but that only about 20% of the cytosines in CpG dinucleotides is methylated in female DNA (Figure 2). Since CpG island-containing promoters are usually highly methylated on the inactive X and unmethylated on the active X, analysis of female DNA usually shows approximately 50% methylation of CpG islands of genes subject to XCI. This is the net result of approximately100% methylation of genes on the inactive X and their complete lack of methylation on the active X. Analysis of male DNA shows only the pattern of the active X—the absence of methylation [27]. Thus the remarkably low methylation level of this HC promoter is consistent with the results from expression of the *HSD17B10* gene in hybrids showing escape from XCI [16,28,29].

Chromosomal DNA isolated from blood is widely used for the methylation analysis of X-linked promoters as leukocytes are non-dividing cells with a short life span [31]. Since it was reported that at least 88% of human genes do not show tissue-specific XCI [27], data obtained from blood chromosomal DNA generally represents the XCI status in other tissues. This is true for the gene of interest, because the methylation profile of the *HSD17B10* proximal promoter shown in Figure 2 was found to be like that of human dorsolateral prefrontal cortex according to the methylomeDB, a database of DNA methylation profiles of the brain [32]. Hypomethylation of this promoter was found in both blood and brain. Although it was previously reported in a study of fibroblasts [33,34] that the *HSD17B10* gene did not escape XCI, the opposite inference [16] is implied by the data presented here. Data from epigenetic analysis of the *HSD17B10* proximal promoter is consistent with a variable escape of this gene from XCI. This suggests that some bi-allelic expression of this gene may occur in female cells, which could add to the variability in expression imposed by mosaicism of XCI [35]. It is conceivable that the variable escape may allow more cells to have at least some wild type HSD10 in a female carrying a missense mutation on the *HSD17B10* gene.

Missense mutations of the *HSD17B10* gene result in an X-linked metabolic disease in males while most females are asymptomatic [36]. So far only two female cases with severe developmental disabilities have been reported [37,38]. More interestingly, female patients uniformly present milder clinical manifestations than male patients carrying the same mutations. The variable escape of the *HSD17B10* gene from XCI may provide another protective factor for a female for alleviation of HSD10 deficiency.

## Conclusions

The actual length of first exon of the HSD17B10 gene was found to be about a quarter larger than that originally reported. Its transcripts result from a slippery transcription complex. The hypomethylation of the CpG island in the proximal promoter of the HSD17B10 gene provides additional evidence for the variable escape of the HSD17B10 gene from X-chromosome inactivation which could influence the range of severity of HSD10 deficiency, an inherited error in isoleucine metabolism, in heterozygous females.

## Competing interests

The authors declare that they have no competing interests.

## Authors’ contributions

SYY, CD and WTB conceived the study. XYH, CD and SYY performed experiments. SYY and CD drafted the manuscript. All authors have read and approved the final manuscript.

## Authors’ information

SYY: MD, PhD, email: songyu.yang@csi.cuny.edu, Head of the Medical Biochemistry Lab;

CD: PhD, email: Carl.Dobkin@opwdd.ny.gov, Head of the Molecular Genetics Lab;

XYH: PhD, email: xue-ying.he@opwdd.ny.gov, Senior Scientist;

WTB: MD, PhD, ted.brown@opwdd.ny.gov, Director of the NYSIBRDD.

## Supplementary Material

Additional file 1 ASSAY DESIGN REPORT of the EpigenDx Inc. Click here for file

Additional file 2 **The dideoxy sequencing ladder: C, cytosine; T, thymine; A, adenine; G, guanosine; M, pBR322 DNA *MspI *Digest.** Click here for file
