# Supplementary material for: Transcription start sites and epigenetic analysis of the HSD17B10 proximal promoter
Source: BMC Biochem. 2013 Jul 8;14:17. doi: 10.1186/1471-2091-14-17 (PMC3729668; doi:10.1186/1471-2091-14-17)
Supplement: Additional file 1 — ASSAY DESIGN REPORT of the EpigenDx Inc. [file 1471-2091-14-17-S1.doc]

1. Intent of Use [2](#__RefHeading___Toc315161328)

2. Summary [2](#__RefHeading___Toc315161329)

3. Human HSD17B10 CpG Methylation Assays [3](#__RefHeading___Toc315161330)

3.1. Materials Provided: [3](#__RefHeading___Toc315161331)

3.2. Materials required but not included [3](#__RefHeading___Toc315161332)

3.3. Human HSD17B10 Promoter and Surrounding Genomic DNA Sequence [3](#__RefHeading___Toc315161333)

3.4. Bisulfite converted target sequences: [4](#__RefHeading___Toc315161334)

3.4.1. ADS2502 [4](#__RefHeading___Toc315161335)

3.4.2. ADS2501 [4](#__RefHeading___Toc315161336)

3.5. Target CpG Sites Coordinates [5](#__RefHeading___Toc315161337)

3.6. Assay Target Sequences [5](#__RefHeading___Toc315161338)

4. Assay Validation [6](#__RefHeading___Toc315161339)

4.1. Selected Assay Validation Results [6](#__RefHeading___Toc315161340)

4.1.1 ADS2502FS1 Validated by Pyrosequencing [6](#__RefHeading___Toc315161341)

4.1.2 ADS2502FS2 Validated by Pyrosequencing [7](#__RefHeading___Toc315161342)

4.1.3 ADS2501FS1 Validated by Pyrosequencing [8](#__RefHeading___Toc315161343)

4.2. PCR Bias Testing Using Pyrosequencing [9](#__RefHeading___Toc315161344)

4.3. Example of Quantitative Analysis (in % Methylation) [9](#__RefHeading___Toc315161345)

5. Protocols [10](#__RefHeading___Toc315161346)

5.1. Assay Template [10](#__RefHeading___Toc315161347)

5.1.1 Template [10](#__RefHeading___Toc315161348)

5.1.2 Bisulfite modification [10](#__RefHeading___Toc315161349)

5.2 PCR Protocol [10](#__RefHeading___Toc315161350)

5.2.1 Recommended PCR protocol [10](#__RefHeading___Toc315161351)

5.2.2 Recommended PCR cycling conditions: [10](#__RefHeading___Toc315161352)

6. Pyrosequencing^TM^ analysis [11](#__RefHeading___Toc315161353)

6.1. Pyrosequencing Protocol: [11](#__RefHeading___Toc315161354)

Explanations and abbreviations:

**Tail**: A short tail (*eg.* TTT) may have been added to the 5’-end of the non-biotinylated PCR primer in order to reduce the possibility of self-priming in the PCR product.

**Abbreviations**: F=forward, R=reverse, P=PCR primer, S=sequencing primer, B=biotin.

# Intent of Use

HSD17B10 encodes 3-hydroxyacyl-CoA dehydrogenase type II, a member of the short-chain dehydrogenase/reductase superfamily. The gene product is a mitochondrial protein that catalyzes the oxidation of a wide variety of fatty acids, alcohols, and steroids. The protein has been implicated in the development of Alzheimer's disease, and mutations in the gene are the cause of 2-methyl-3-hydroxybutyryl-CoA dehydrogenase deficiency (MHBD). Several alternatively spliced transcript variants have been identified, but the full-length nature of only two transcript variants has been determined. [provided by RefSeq, Jul 2008]. This gene is located at [Chromosome X: 53,458,206-53,461,320](http://www.ensembl.org/Homo_sapiens/Location/View?db=core;g=ENSG00000072506;r=X:53458206-53461320) reverse strand. There are 5 transcriptional variants of this gene.

This assay was designed for the analysis of DNA methylation. It is for research use only and is NOT to be used in diagnostic procedures. EpigenDx takes no responsibility for the use of these reagents in any other context than specified in this manual.

# Summary

| **Assay ID** | **Gene Name** | **CpG Loci** | **Also Known As** | **Type of assay** | **PCR Size** | **# of CpGs** |
| --- | --- | --- | --- | --- | --- | --- |
| ADS2502 | Human hydroxysteroid (17-beta) dehydrogenase 10  Ensembl Gene ID:  ENSG00000072506 | Human HSD17B10  Ensembl Transcript ID:  [ENST00000168216](http://www.ensembl.org/Homo_sapiens/Transcript/Exons?db=core;g=ENSG00000072506;r=X:53458206-53461320;t=ENST00000168216)  -171 to -7 from ATG  -143 to +22 from TSS | ABAD; CAMR; ERAB; HCD2; MHBD; HADH2; MRPP2; MRX17; MRX31; SCHAD; MRXS10; SDR5C1; 17b-HSD10; DUPXp11.22 | Bisulfite sequencing | 217 bp | 22 |
| ADS2501 | Human hydroxysteroid (17-beta) dehydrogenase 10  Ensembl Gene ID:  ENSG00000072506 | Human HSD17B10  Ensembl Transcript ID:  [ENST00000168216](http://www.ensembl.org/Homo_sapiens/Transcript/Exons?db=core;g=ENSG00000072506;r=X:53458206-53461320;t=ENST00000168216)  +2259 from ATG  +2287 from TSS |  | Bisulfite sequencing | 240 bp | 1 |

# Human HSD17B10 CpG Methylation Assays

## Materials Provided:

| **Assay ID** | **CpG Loci** | **Type of primer** | **Primer name** | **5' mod** |
| --- | --- | --- | --- | --- |
| ADS2502 | Human HSD17B10  Ensembl Transcript ID:  ENST00000168216  -171 to -7 from ATG  -143 to +22 from TSS | PCR F | ADS2502FP |  |
|  |  | PCR R | ADS2502RPB | biotin |
| ADS2502FS1 | CpG #1 to #3 | Seq F | ADS2502FS1 |  |
| ADS2502FS2 | CpG #4 to #23 | Seq F | ADS2502FS2 |  |
| ADS2501 | Human HSD17B10  Ensembl Transcript ID:  ENST00000168216  +2259 from ATG  +2287 from TSS | PCR F | ADS2501FP |  |
|  |  | PCR R | ADS2501RPB | Biotin |
| ADS2501FS1 | CpG #1 | Seq F | ADS2501FS1 |  |

## Materials required but not included

- DNA extraction reagents.

- PCR reagents

- Pyrosequencing or downstream analysis reagents and consumables.

## Human HSD17B10 Promoter and Surrounding Genomic DNA Sequence

ctgtactgtaattctctttctccacctggactgtgccctcctggaaggcatgaatcttttattattccgctgggtatcccaggtgccccgcacaccgtctagcatataggagtctcaaatacattcatacttgaattaagactttttcagaggagaaaggcatccgttctcaatatccacccctcttcccccacgcggagcaggccacttgccaaaaggccagctagagagtagggcgaggggtgttgtggtattgtacttctgctattggtctctttccccagttgaaataactctggggataccttagcaggcggaatccgccctctggccaaaggactagcgtaccaggccacgcccccacgtctcatgcggcagcggcagacgccccggcccgtcgcatccgccccttccgccg[c](http://www.ensembl.org/Homo_sapiens/ZMenu/TextSequence?db=core;factorytype=Location;g=ENSG00000072506;r=X:53458206-53461320;t=ENST00000168216;v=rs1264014;vf=18152394)ttcgcctcggccaatcaacgagcgcccgcgcccccatcCCCATCCCGTGGAGTGGCCGGCGACAAGATGGCAGCAGCGTGTCGGAGCGTGAAGgta[g](http://www.ensembl.org/Homo_sapiens/ZMenu/TextSequence?db=core;factorytype=Location;g=ENSG00000072506;r=X:53458206-53461320;t=ENST00000168216;v=rs112303075;vf=19346461)aaggttctttgcctctgttgcttgccttcctttacgggccgggacagcgcggccgtggaactaggagaaagagatcggtttcgagggaaagggtcatgcttatgtaggcctggtattgtggccgttgggctcaagaggtgaaagtcagcactccgcggcgcatctgtctgtctgtctgtctgtctatctatctatctatcaatcatctatctatctatcgtcacgggacgggctgtctggggatgagatcccttctgcccccgaggtcccaacgctgaggagaagcag[c](http://www.ensembl.org/Homo_sapiens/ZMenu/TextSequence?db=core;factorytype=Location;g=ENSG00000072506;r=X:53458206-53461320;t=ENST00000168216;v=rs139681190;vf=19516388)acacctagtttacccgcaggggtgaccacgacctctcccctccccc[c](http://www.ensembl.org/Homo_sapiens/ZMenu/TextSequence?db=core;factorytype=Location;g=ENSG00000072506;r=X:53458206-53461320;t=ENST00000168216;v=rs12851009;vf=18500143)ttctcctca[g](http://www.ensembl.org/Homo_sapiens/ZMenu/TextSequence?db=core;factorytype=Location;g=ENSG00000072506;r=X:53458206-53461320;t=ENST00000168216;v=rs12852645;vf=18501600)aagcctgga[g](http://www.ensembl.org/Homo_sapiens/ZMenu/TextSequence?db=core;factorytype=Location;g=ENSG00000072506;r=X:53458206-53461320;t=ENST00000168216;v=rs12846968;vf=18496608)aacaggcaggcg[a](http://www.ensembl.org/Homo_sapiens/ZMenu/TextSequence?db=core;factorytype=Location;g=ENSG00000072506;r=X:53458206-53461320;t=ENST00000168216;v=rs12854137;vf=18502621)cagcg[t](http://www.ensembl.org/Homo_sapiens/ZMenu/TextSequence?db=core;factorytype=Location;g=ENSG00000072506;r=X:53458206-53461320;t=ENST00000168216;v=rs12847845;vf=18497392)ggggatggtctgggcaatgtaacagctgaccctgaccatgtcccttttgacagGGCCTGG[T](http://www.ensembl.org/Homo_sapiens/ZMenu/TextSequence?db=core;factorytype=Location;g=ENSG00000072506;r=X:53458206-53461320;t=ENST00000168216;v=rs12847788;vf=18497340)GGCGGTAATAACCGGAGGAGCCTCGGGCCTGGGCCTGGCCACGGCGGAGCGACTTGTGGGGC[A](http://www.ensembl.org/Homo_sapiens/ZMenu/TextSequence?db=core;factorytype=Location;g=ENSG00000072506;r=X:53458206-53461320;t=ENST00000168216;v=rs144679727;vf=19671262)GGGAGCCTCTGCTGTGCTTCTGGACCTGCCCAACTCGGGTGGGGAGGCCCAAGCCAAGAAGTTAGGAAACAACTG[C](http://www.ensembl.org/Homo_sapiens/ZMenu/TextSequence?db=core;factorytype=Location;g=ENSG00000072506;r=X:53458206-53461320;t=ENST00000168216;v=rs140876830;vf=19553380)GTTTTCGCCCCAGCCGACgtaagtggggtcacctctcctctcccagggtgtgcatgaggtcaccaacaccggcctgggaacctgtgggggtccctcccataagggtctcctctatgggggcggtagacccatctccttgtctcttttggcctgagccttcaagcactgtgggaccacctgtgataaggattctctaaagctgggattcacaacaatgaggatggggattggac[g](http://www.ensembl.org/Homo_sapiens/ZMenu/TextSequence?db=core;factorytype=Location;g=ENSG00000072506;r=X:53458206-53461320;t=ENST00000168216;v=rs146366586;vf=19723306)taacaatctcctggaggagttatgtatggtttgaaaatgcattttgtgccttgtaattttatatttacaaagtcaaaaaacttgttttcatgaaacaaactacagaaagtgaaacttgatgaaatcatcttagcaggtaaggatacaaaaaagctagtttatcttcctatcaataattctcacaa[g](http://www.ensembl.org/Homo_sapiens/ZMenu/TextSequence?db=core;factorytype=Location;g=ENSG00000072506;r=X:53458206-53461320;t=ENST00000168216;v=rs147949967;vf=19772246)agggttattttttatttttatttttgagatggagtctcgctctgttgcccaggctggagtgcagtggcacaatctcagctccttgcaacctccatcttctgggttcaagcgattttcctgcctcaacctcccaagtagctgggattacaggcacctgccaccacgcccagctaatttttttttttgtatttttagaagagatggggtttcaccatgttggccgggctggtcttgaactcctgatctcacgtgatccgcccgcctcggcctcccaaagtgctgggattacaggtgtgagccactgcaccc[a](http://www.ensembl.org/Homo_sapiens/ZMenu/TextSequence?db=core;factorytype=Location;g=ENSG00000072506;r=X:53458206-53461320;t=ENST00000168216;v=rs141583477;vf=19575219)gcctcacaacagggttgcattagtgctctgatccacagcaattaagaatcttcaggagcatgtgaatttatcaagaaggtattgatttgaaaaatgttttgcaacactgctctatattacctaatattttcacctttgacctactctctcaatttttttttttgggacagagtctcactgttgcccaggctgcag[t](http://www.ensembl.org/Homo_sapiens/ZMenu/TextSequence?db=core;factorytype=Location;g=ENSG00000072506;r=X:53458206-53461320;t=ENST00000168216;v=rs138479036;vf=19479085)gcaatggcacgatctcagctcactgcaacctctgcctcccgggttcaagcgattcttctgcctcagccttccaagtagctgggattacaggcatgtgtcactatgccggctaattttgtatttttagtagagacgggttttcaccacgttggccaggctggtctcaaactcctgatcttaagtgatccaccagcctcggcctcccaaagtgctgagattacaggcatgagccaccgcacccagcctatctctcaagttttacaacctttctcttggcctctcccttctcacaaatctcttctcacccaaggactgggcatgccctgccctgccctccctcaggtttagtgggaagagtgggtgctgggagatgaataccttctccacttctctctttgaagG[T](http://www.ensembl.org/Homo_sapiens/ZMenu/TextSequence?db=core;factorytype=Location;g=ENSG00000072506;r=X:53458206-53461320;t=ENST00000168216;v=rs104886492;vf=19278717)GACCTCTGAGAAGGATGTGCAAACAGCTCTGGCTCTAGCAAAAGGAAAGTTTGGCCGTGTGGATGTAGCTGTCAACTGTGCAGGCATCGCGGTGGCTAGCAAGACGTACAACTTAAAGAAGGGCCAGACCCATACCTTGGAAGACTTCCAGCGAGTTCTTGATgtaaggccttggaagttctccagggatagtggtagtaagaagtatctggctgtggaggaccccaaagtttttaggggacaaaggcttcttctgcctcaggtcctatagcatgtctccatctgtccta[c](http://www.ensembl.org/Homo_sapiens/ZMenu/TextSequence?db=core;factorytype=Location;g=ENSG00000072506;r=X:53458206-53461320;t=ENST00000168216;v=rs112756915;vf=19374405)agGTGAAT[C](http://www.ensembl.org/Homo_sapiens/ZMenu/TextSequence?db=core;factorytype=Location;g=ENSG00000072506;r=X:53458206-53461320;t=ENST00000168216;v=CM031196;v=rs28935476;vf=44663077;vf=18537689)TCATGGGCACCTTCAATGTGATC[C](http://www.ensembl.org/Homo_sapiens/ZMenu/TextSequence?db=core;factorytype=Location;g=ENSG00000072506;r=X:53458206-53461320;t=ENST00000168216;v=CM031197;v=rs28935475;vf=44663076;vf=18537688)GCCTGGTGGCTGGTGAGATGGGCCAGAATGAACCAGACCAGGGAGGCCAACGTGGGGTCATCATCAACACTGCCAGTGTGGCTGCCTTCGAGGGTCAGgtgtgtgggcaggggtaagacttatgcctcctaagtgacttgttggggccctcccacctatgacttctactctttctccagGTTGGACAAGCTGCATACTCTGCTTCCAAGGGGGGAATAGTGGGCATGACACTGCCCATTGCTCGGGATCTGGCTCCCATAGGTATC[C](http://www.ensembl.org/Homo_sapiens/ZMenu/TextSequence?db=core;factorytype=Location;g=ENSG00000072506;r=X:53458206-53461320;t=ENST00000168216;v=rs122462164;v=CM070155;vf=19458949;vf=44663075)GGGTGATGACCATTGCCCCAGgtagacatatcccctctctccatcatacctgggattgggtgggatccatggacagttga[g](http://www.ensembl.org/Homo_sapiens/ZMenu/TextSequence?db=core;factorytype=Location;g=ENSG00000072506;r=X:53458206-53461320;t=ENST00000168216;v=rs12688475;vf=18481188)aggggaaggtatccaccacctaagcagcagcagccttttatctttgggccccagagaagcaccaa

- Lower cases letters in green are 5’ upstream sequence.
- Lower case letters in blue are the intron sequence.
- Capital letters in purple are the exon 1 (5’UTR) sequence.
- Capital letters in black are the exon 1 coding region
- CpG sites in this sequence are red (CGs).
- Green highlighted (C) is the transcriptional start site.
- Green highlighted (ATG) is the translational start site (ATG).
- ADS2501 target CpG loci are blue highlighted (CG).
- ADS2502 target CpG loci are gray highlighted (CG).

## Bisulfite converted target sequences:

### ADS2502

taggyggaattygttttttggttaaaggattagygtattaggttaygtttttaygttttatgyggtagyggtagaygtttyggttygtygtattygtttttttygtyg[t](http://www.ensembl.org/Homo_sapiens/ZMenu/TextSequence?db=core;factorytype=Location;g=ENSG00000072506;r=X:53458206-53461320;t=ENST00000168216;v=rs1264014;vf=18152394)ttygtttyggttaattaaygagygttygygtttttattTTTATTTYGTGGAGTGGTYGGYGATAAGATGGTAGTAGYGTGT

### ADS2501

T[T](http://www.ensembl.org/Homo_sapiens/ZMenu/TextSequence?db=core;factorytype=Location;g=ENSG00000072506;r=X:53458206-53461320;t=ENST00000168216;v=CM031197;v=rs28935475;vf=44663076;vf=18537688)GTTTGGTGGTTGGTGAGATGGGTTAGAATGAATTAGATTAGGGAGGTTAAYGTGGGGTTATTATTAATATTGTTAGTGTGGTTGTTTTYGAGGGTTAGgtgtgtgggtaggggtaa

## Target CpG Sites Coordinates

|  | **ADS2502** | **ADS2501** |
| --- | --- | --- |
| **From ATG** | -171 to -7 | +2259 |
| **From TSS** | -143 to +22 | +2287 |
| **GCRh36/hg18 X Chr** | 53478188 to 53478024 | 53459035 |
| **GCRh37/hg19 X Chr** | 53461463 to 53461299 | 53475759 |

## Assay Target Sequences

| **Assay ID** | **Genomic Target Sequence** | **Bisulfite Converted Target Sequence** | **Pyrosequencing Dispensation order** |
| --- | --- | --- | --- |
| ADS2502FS1 | caggcggaatccgccctctggccaaaggactagcgtaccagg | taggyggaattygttttttggttaaaggattagygtattagg | ATCATGTCGTATCGTCTGCTAGACTATGTCGTA |
| ADS2502FS2 | ccacgcccccacgtctcatgcggcagcggcagacgccccggcccgtcgcatccgccccttccgccg[c](http://www.ensembl.org/Homo_sapiens/ZMenu/TextSequence?db=core;factorytype=Location;g=ENSG00000072506;r=X:53458206-53461320;t=ENST00000168216;v=rs1264014;vf=18152394)ttcgcctcggccaatcaacgagcgcccgcgcccccatcCCCATCCCGTGGAGTGGCCGGCGACAAGATGG ([c](http://www.ensembl.org/Homo_sapiens/ZMenu/TextSequence?db=core;factorytype=Location;g=ENSG00000072506;r=X:53458206-53461320;t=ENST00000168216;v=rs1264014;vf=18152394)>g, [rs1264014](http://useast.ensembl.org/"/Homo_sapiens/Variation/Summary?db=core;g=ENSG00000072506;r=X:53458206-53461320;source=dbSNP;t=ENST00000168216;v=rs1264014;vf=18152394\")) | ttaygtttttaygttttatgyggtagyggtagaygtttyggttygtygtattygtttttttygtyg[t](http://www.ensembl.org/Homo_sapiens/ZMenu/TextSequence?db=core;factorytype=Location;g=ENSG00000072506;r=X:53458206-53461320;t=ENST00000168216;v=rs1264014;vf=18152394)/gttygtttyggttaattaaygagygttygygtttttattTTTATTTYGTGGAGTGGTYGGYGATAAGATGG | TCGATCGTCTGATCGTCTATAGTCGTCATGTCGTAGTATCAGTTCGTCAGTCGTCGATCGTTCAGTCGTTCTGTTCGTCATCGATCGATGTCAGTCGTCGTCTATTGATTCGTGAGTAGTCGTCGA |
| ADS2501FS1 | TC[C](http://www.ensembl.org/Homo_sapiens/ZMenu/TextSequence?db=core;factorytype=Location;g=ENSG00000072506;r=X:53458206-53461320;t=ENST00000168216;v=CM031197;v=rs28935475;vf=44663076;vf=18537688)GCCTGGTGGCTGGTGAGATGGGCCAGAATGAACCAGA ([C](http://www.ensembl.org/Homo_sapiens/ZMenu/TextSequence?db=core;factorytype=Location;g=ENSG00000072506;r=X:53458206-53461320;t=ENST00000168216;v=CM031197;v=rs28935475;vf=44663076;vf=18537688)>T, [rs28935475](http://useast.ensembl.org/"/Homo_sapiens/Variation/Summary?db=core;g=ENSG00000072506;r=X:53458206-53461320;source=dbSNP;t=ENST00000168216;v=rs28935475;vf=18537688\") lost CpG status) | T[Y](http://www.ensembl.org/Homo_sapiens/ZMenu/TextSequence?db=core;factorytype=Location;g=ENSG00000072506;r=X:53458206-53461320;t=ENST00000168216;v=CM031197;v=rs28935475;vf=44663076;vf=18537688)GTTTGGTGGTTGGTGAGATGGGTTAGAATGAATTAGA | ATCGCTGTGCTG |

# Assay Validation

## Selected Assay Validation Results

### ADS2502FS1 Validated by Pyrosequencing

**Sequence to analyze:**

TAGGYGGAATTYGTTTTTTGGTTAAAGGATTAGYGTATTAGG

(Yellow highlights are the controls for bisulfite modification completion)

**Histogram:**

**Pyrogram:**

**Control: Low methylated DNA**

**Control: Medium methylated DNA**

**Control: High methylated DNA**

**Control: No template control**

### ADS2502FS2 Validated by Pyrosequencing

**Sequence to analyze:**

TTAYGTTTTTAYGTTTTATGYGGTAGYGGTAGAYGTTTYGGTTYGTYGTATTYGTTTTTTTYGTYGT/GTTYGTTTYGGTTAATTAAYGAGYGTTYGYGTTTTTATTTTTATTTYGTGGAGTGGTYGGYGATAAGATGG (Yellow highlights are the controls for bisulfite modification completion)

**Histogram:**

**Pyrogram:**

**Control: Low methylated DNA**

**Control: Medium methylated DNA**

**Control: High methylated DNA**

**Control: No template control**

### ADS2501FS1 Validated by Pyrosequencing

**Sequence to analyze:**

TYGTTTGGTGGTTGGTGAGATGGGTTAGAATGAATTAGA

(Yellow highlights are the controls for bisulfite modification completion)

**Histogram:**

**Pyrogram:**

**Control: Low methylated DNA**

**Control: Medium methylated DNA**

**Control: High methylated DNA**

**Control: No template control**

## PCR Bias Testing Using Pyrosequencing

Unmethylated DNA control and in vitro methylated DNA were mixed at different ratios followed by bisulfite modification, PCR, and Pyrosequencing analysis. The percent methylation obtained from the mixing study should be highly correlated with expected methylation percentages with an r-square of 0.8 or higher.


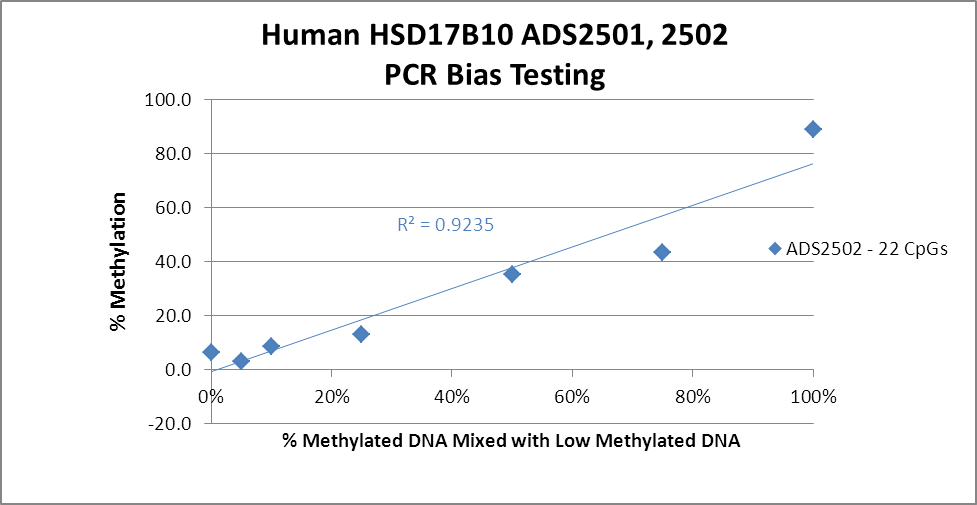


## Example of Quantitative Analysis (in % Methylation)

# **Protocols**

## Assay Template

### Template

EpigenDx in-house control DNA was used for the assay development.

### Bisulfite modification

Bisulfite modification was carried out using Zymo Research EZ Methylation kit (Cat.#D5002 or D5004). 200 - 500 ng of sample DNA was used for bisulfite modification followed by the PCR amplification.

## PCR Protocol

### Recommended PCR protocol

We recommend using HotStar Taq Polymerase Qiagen Cat.#203205. Additional optimization is needed if different PCR system is used in analysis.

**ADS2502**

| **Component** | **Per 30μl reaction** |
| --- | --- |
| 10X PCR buffer (Contains 15mM MgCl_2_) | 3 μl (1x) |
| 25 mM MgCl_2_ | 1.8 μl (3.0 mM final conc.) |
| 10 mM dNTPs | 0.6 μl (200 μM of each) |
| 10 μM Fwd primer (ADS2502FP) | 0.6 μl (6 pmol) |
| 10 uM Rev primer (ADS2502RPB) | 0.6 ul (6 pmol) |
| HotStar Taq Polymerase (5 U/μl) | 0.15 μl (0.75 U) |
| DNA | 1 µl of bisulfite treated DNA |
| Water | Adjust to 30 µl |

**ADS2501**

| **Component** | **Per 30μl reaction** |
| --- | --- |
| 10X PCR buffer (Contains 15mM MgCl_2_) | 3 μl (1x) |
| 25 mM MgCl_2_ | 1.8 μl (3.0 mM final conc.) |
| 10 mM dNTPs | 0.6 μl (200 μM of each) |
| 10 μM Fwd primer (ADS2501FP) | 0.6 μl (6 pmol) |
| 10 uM Rev primer (ADS2501RPB) | 0.6 ul (6 pmol) |
| HotStar Taq Polymerase (5 U/μl) | 0.15 μl (0.75 U) |
| DNA | 1 µl of bisulfite treated DNA |
| Water | Adjust to 30 µl |

### Recommended PCR cycling conditions:

***ADS2502***

95ºC 15 min; 45 x (95ºC 15s; **53**ºC 30 s; 72ºC 30 s); 72ºC 5 min; 4°C ∞

***ADS2501***

95ºC 15 min; 45 x (95ºC 15s; **60**ºC 30 s; 72ºC 30 s); 72ºC 5 min; 4°C ∞

# Pyrosequencing^TM^ analysis

The PSQ™96HS system should be used according to standard procedures with the dispensation orders assigned for each assay as stated in this report. Additional optimization may be needed if different analysis system is used.

## Pyrosequencing Protocol:

Pyrosequencing is performed using PSQ 96HS system or PSQ 96HSA system. The systems are equivalent to the PyroMark MD system. Pyrosequencing analysis is performed as per manufactory’s recommended protocol with modification:

1. Prepare master Binding Solution:

- Components for 1 reaction of binding solution
  - - - 2.2 μl streptavidin
      - 40 μl of 2X Binding Buffer
      - 25 μl Milli-Q-water

1. Add 65 μl of binding solution to each 15 μl PCR sample.
2. Capture the PCR product as manufactory’s protocol.
3. Release the sepherose beads into annealing buffer containing 0.5 μM of a sequencing primer.
4. Anneal the sequencing primer to the template by heating the plate to 85 ºC for 2 minutes.
5. Turn off the heating block and leave the Pyro plate on the heating block for 10 minutes.
6. Remove the Pyro plate from the heating block and allow the plate continuing to cool for 5 minutes.
7. Run the Pyrosequencing as the manufactory’s instruction.

EpigenDx Assay Design Service (ADS)

General Terms and Conditions

**1. Application**

These general terms and conditions are applicable to all of EpigenDx’s services. Every Customer placing an order with EpigenDx accepts these general conditions prior to placing the order.

**2. Orders**

Orders will only be acted upon if confirmed by a written acceptance from Pyrosequencing.

**3. Price**

EpigenDx’s prices are quoted in US dollar and are exclusive of VAT. Prices may be subject to change at any time.

**4. Delivery times**

EpigenDx will use reasonable efforts to deliver results within the period of time agreed with the Customer.

However, agreed delivery times are not binding and delays in delivery shall not give rise to cancellation of the order.

**5. Shipment of samples**

When samples from the Customer are necessary in order to perform the assay, the Customer shall secure express approval from EpigenDx to ship the samples to be analyzed by EpigenDx. The Customer shall appropriately pack and label the samples and pay for the shipment. EpigenDx shall not be liable for loss or damage of the samples during shipment, storage or use while performing the ADS. After performing the services, EpigenDx may, at its option, decide to return to the Customer or to destroy any remaining samples. EpigenDx reserves the right to refuse material that is hazardous, controlled or prohibited by law.

**6. Confidentiality**

EpigenDx will use all reasonable endeavors to keep in strict confidence any information and results, related to an order by the Customer. Only the Customer will be allowed to access such information and results, except if EpigenDx is required by law to disclose information or results.

**7. Intellectual Property Rights**

The Customer shall have the unrestricted right to use any and all results, from their order. EpigenDx shall own all right, title and interest in and to any technology, methods, assay designs, know-how and any inventions used by EpigenDx to perform the service for the Customer.

**8. Limited warranty**

EpigenDx shall use all reasonable efforts to perform the services with utmost care and skill.

EpigenDx makes no other Warranty, express or implied, including any warranty of merchantability, title, or fitness for a particular use.

In particular, EpigenDx does not warrant that the use of results will not infringe intellectual property rights of third parties.

**9. Limitation of liability**

After delivery of the results and material to the Customer, EpigenDx assumes no further liability than that provided under Article 8.

In no event shall EpigenDx be held liable for any damages arising from loss of earnings or any other loss arising directly or indirectly from the use of the results.

The Customer agrees to assume any and all liability for the Customer’s use of the results and to hold EpigenDx harmless from any claims or liabilities that might arise from the Customer’s use of the results and material.

**10. Payment**

Invoices are due and payable within thirty calendar days of the invoice date unless otherwise agreed with EpigenDx. Late payments will be charged 1% interest per month.

**11. Termination**

EpigenDx shall have the unilateral right to cancel any order without being liable for any damages, by notifying the Customer of EpigenDx’s intention through e-mail.

**12. Applicable Law**

This agreement will be governed and interpreted in accordance with US law.
