# Supplementary material for: Transcription start sites and epigenetic analysis of the HSD17B10 proximal promoter
Source: BMC Biochem. 2013 Jul 8;14:17. doi: 10.1186/1471-2091-14-17 (PMC3729668; doi:10.1186/1471-2091-14-17)
Supplement: Additional file 2 — The dideoxy sequencing ladder: C, cytosine; T, thymine; A, adenine; G, guanosine; M, pBR322 DNA MspI Digest. [file 1471-2091-14-17-S2.docx]

**SUPPLEMENTAL MATERIAL 2**


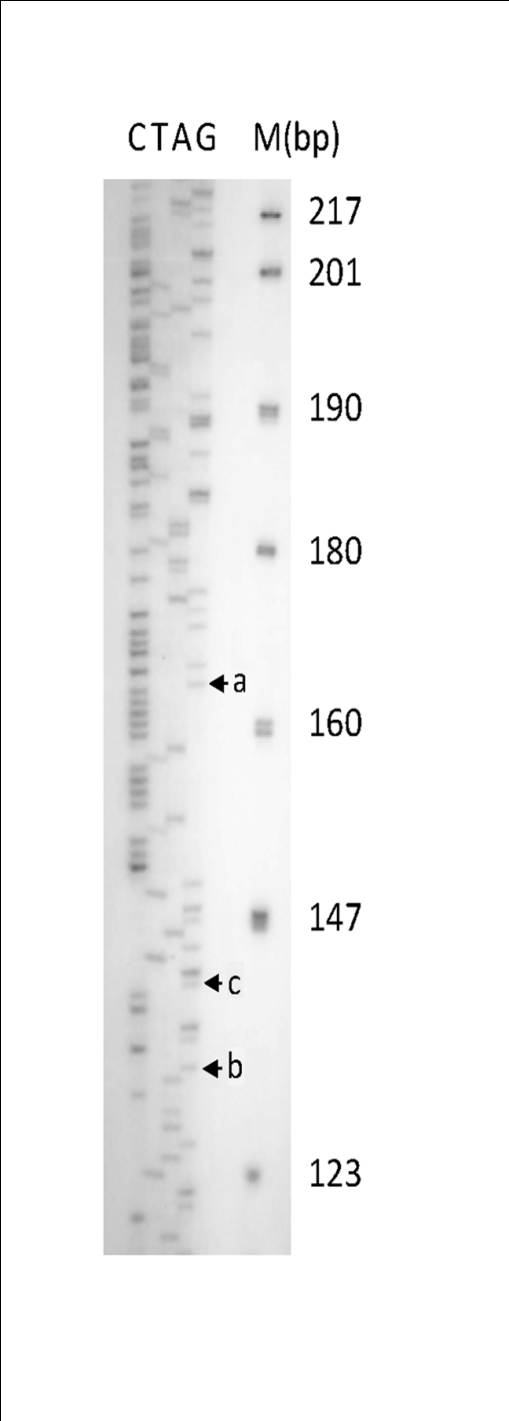
The dideoxy sequencing ladder: C, cytosine; T, thymine; A, adenine; G, guanosine; M, pBR322 DNA *MspI* Digest.
